# The efficacy of Sarcomeal® oral supplementation plus vitamin D3 on muscle parameters, metabolic factors, and quality of life in diabetic sarcopenia: a randomized controlled clinical trial

**DOI:** 10.1007/s40520-025-02969-x

**Published:** 2025-03-13

**Authors:** Ramin Abdi Dezfouli, Narges Zargar Balajam, Ramin Heshmat, Gita Shafiee

**Affiliations:** 1 Chronic Diseases Research Center, Endocrinology and Metabolism Population Sciences Institute, Tehran University of Medical Sciences, Tehran, Iran; 2 Endocrinology and Metabolism Research Center, Endocrinology and Metabolism Clinical Sciences Institute, Tehran University of Medical Sciences, Tehran, Iran

**Keywords:** Type 2 diabetes, Sarcopenia, Intervention, Protein supplement, Vitamin D, Quality of life

## Abstract

**Aim:**

To investigate the efficacy of Sarcomeal® sachet, as a protein supplement, plus vitamin D3 on muscle parameters, metabolic factors, and quality of life (QoL) in individuals with diabetes and sarcopenia.

**Methods:**

Sixty individuals were randomized into the control or intervention group. The intervention group received a daily dose of one Sarcomeal sachet and 1000 IU of vitamin D and both groups were recommended to consume protein-rich food, be educated about the disease, and perform physical activity for 12 weeks. Various assessments including muscle parameters, blood tests, and QoL were conducted at the beginning and the end of the trial.

**Results:**

Over 12 weeks, although the intervention group had significant improvements in mean skeletal muscle mass index (SMI) (change: 0.17[0.016, 0.329] kg/m²; *p* < 0.05) and handgrip strength (change: 1.33[0.256, 2.410] kg; *p* < 0.05), differences between groups were not statistically significant. However, significant improvements were observed in lean mass (1.70 [0.749, 2.665] kg; *P* < 0.01) and lean mass index (0.62[0.287, 0.954] kg/m^2^; *P* < 0.01) between groups. Weight was maintained in the intervention arm, whereas the control arm experienced significant weight loss (1.87 [0.654, 3.109] kg; *P* < 0.01). Participants in the intervention arm did not show significant changes in blood parameters. The most reported side effects were loss of appetite (50%) and stomach heaviness (20.8%).

**Conclusion:**

This mixture of supplements significantly improved lean muscle mass, preserved physical function, and helped maintain weight, supporting its potential as a strategy to counter muscle loss and enhance the QoL in diabetic sarcopenia patients.

**Clinical trial registration:**

This trial is registered at the Iranian Registry of Clinical Trials (IRCT) with IRCT20230831059311N1 ID.

## Introduction

Sarcopenia, characterized by a decline in muscle mass, strength, and functionality, primarily affects older individuals [1]. In addition to factors like age, physical inactivity, malnutrition, smoking, and disrupted sleep patterns, sarcopenia commonly occurs as a complication of diabetes [2]. Known as Diabetic Sarcopenia (DS), this condition is characterized by signs of sarcopenia in individuals with diabetes, often resulting from metabolic changes, insulin resistance, and inactivity associated with the disease. The prevalence of DS is estimated to be 18% among diabetic patients [3, 4], posing risks such as impaired physical abilities, increased risk of falls, fractures, and other complications associated with reduced muscle function [5].

DS presents unique metabolic challenges that differentiate it from primary sarcopenia. One of the major challenges in managing DS lies in the metabolic and physiological peculiarities of diabetes, which can exacerbate the decline in muscle function [2]. For instance, diabetes often leads to increased insulin resistance, which impairs glucose uptake in muscle cells and reduces the efficiency of muscle protein synthesis [6]. Moreover, chronic hyperglycemia in diabetes patients can result in the formation of advanced glycation end-products (AGEs), which can damage muscle proteins and other structural components [7]. Additionally, diabetes-related oxidative stress and inflammation can further increase muscle degradation and decrease muscle repair and regeneration [8]. Therefore, managing sarcopenia in diabetic individuals becomes more sophisticated.

The primary approach to managing DS involves lifestyle modifications, with research indicating that well-rounded diets abundant in protein and vital nutrients are crucial for preserving and promoting muscle health [9]. Additionally, incorporating regular resistance training exercises has been shown to enhance muscle mass and strength in individuals with DS [10]. However, sticking to a regular exercise routine can be difficult for older adults, who often deal with age-related physical restrictions, multiple health conditions, and a lack of motivation. Consequently, pharmacological approaches have attracted increasing attention as either an alternative or an addition to exercise routines.

Pharmacological approaches to managing sarcopenia primarily involve hormone replacement therapies like using testosterone, selective androgen receptor modulators (SARMs), and myostatin inhibitors. However, these treatments have not yet been approved by the FDA, and additional research is necessary. Supplements also play an important role in managing sarcopenia, with protein supplements being central to supporting muscle growth and synthesis. Whey protein, extracted from milk, is recognized for its potential benefits in addressing DS [11], owing to its richness in essential amino acids and high biological value. Studies indicate that whey protein supplementation may enhance muscle protein synthesis and increase muscle mass in individuals with diabetes [12], ultimately leading to the prevention or management of DS.

Creatine, branched-chain amino acids (BCAAs), glutamine, hydroxyl-methyl-butyrate (HMB), and vitamin D have also been shown to stimulate muscle protein synthesis, boost muscle mass, enhance muscle performance, and aid in muscle recovery in sarcopenia [13, 14]. While the individual effectiveness of the ingredients mentioned in addressing muscle dystrophies is well-documented, Sarcomeal® (Karen Pharm. Co., Iran) is a supplement specifically formulated for managing sarcopenia, comprising whey protein, creatine, glutamine, BCAAs, and HMB. Consequently, this clinical trial aims to investigate the following question: Does the consumption of Sarcomeal sachets alongside vitamin D lead to improvements in muscle parameters and metabolic factors compared to the control group in patients with DS?

## Methods

This trial is registered at the Iranian Registry of Clinical Trials (IRCT ID: IRCT20230831059311N1), which is recognized as a primary registry within the WHO Registry Network and complies with international standards for clinical trial registries. This study is also approved by the institutional ethics committee of the Endocrine and Metabolism Research Institute, Tehran University of Medical Sciences (IR.TUMS.EMRI.REC.1402.071). The study procedures adhered to the ethical principles outlined in the Declaration of Helsinki for medical research involving human subjects [15]. Participants were provided with both verbal and written explanations regarding the purpose and potential risks associated with the intervention study, aligning with the principles outlined in the Helsinki Declaration. Written informed consent was obtained from all enrolled participants.

### Subjects

Enrollment of 60 subjects began in March 2024 at the Diabetes and Metabolic Diseases Specialist Clinic, Tehran University of Medical Sciences, Iran. For diagnosing sarcopenia among patients, the specified diagnosis cutoffs for identifying sarcopenia among the Iranian healthy population were utilized [16] along with the diagnosis protocol based on the European Working Group on Sarcopenia in Older People (EWGSOP) second edition (2019) [17].

Eligible participants showing signs suggestive of sarcopenia underwent an assessment for the inclusion criteria, which are as follows:

1. Aged 50–75 years.
2. Minimum six-month history of type 2 diabetes mellitus.
3. Absence of severe mobility impairment or musculoskeletal disorders (back pain, knee pain, etc.) that lead to impaired walking or prohibit other measurements.
4. Stable Medical Conditions.
5. Low muscle strength (maximum handgrip strength < 26 kg and < 18 kg for men and women, respectively).
6. Low muscle mass index (Appendicular skeletal muscle mass/ height^2^ < 7 kg/m^2^ and < 5.4 kg/m^2^ for men and women, respectively).

After an initial consultation, giving explanations, and checking the first four inclusion criteria, muscle strength (the fifth inclusion criterion) was assessed using a digital dynamometer device. It is also worth mentioning that the SarsaMod equation, developed by Shafiee et al. (2021) [18], was also used as a tool for predicting the chance of sarcopenia. Individuals failing to meet predefined thresholds for maximum muscle strength in both hands were then given more explanations and in the case that the patient agreed to further measurements, a written consent was obtained. After that, the muscle mass index (the sixth inclusion criterion) was assessed using dual X-ray absorptiometry (DXA) as the final inclusion criterion. In addition to the aforementioned tests, a gait speed test was also carried out.

During the participant selection process, the exclusion criteria were as follows:

1. The existence of comorbid orthopedic and neurological problems such as stroke, cerebral palsy, poliomyelitis, rheumatoid arthritis, and prosthesis.
2. Presence of any foreign dense object in the body that interferes with DXA assessment.
3. Patients with active cancer or recent cancer treatment.
4. History of chronic liver disease.
5. History of chronic kidney disease (CKD).
6. History of gout.
7. Using medications that have drug interactions with the components of the supplement.
8. Individuals with significant cognitive decline or dementia who cannot follow the protocol or give informed consent.
9. Patients currently enrolled in other interventional clinical trials that may interfere with the results.
10. Any known allergies or intolerances to the components of the supplement.
11. Patients not willing to co-operate.

### Procedure

Randomization was performed by permuted block randomization with quadruple blocks. A computer-generated randomization sequence was created by a statistician who was blinded to assignment details and had no involvement in the study. This randomized controlled trial utilized a parallel-group comparison design with an open-label approach. The intervention group received a daily dose of one Sarcomeal sachet along with 1000 IU of vitamin D (Exxon Pharmed Co., Iran) after lunch and both groups were recommended to consume protein-rich food, be educated about the disease, and perform physical activity for 12 weeks. Table 1 shows the detailed components of each Sarcomeal sachet. The exercise recommendation for both groups involved light resistance training, which was provided to patients as a step-to-step exercise brochure. In addition to the brochure, full explanations were also given to all patients for doing exercises.

**Table 1:** Supplement facts per each sachet of sarcomeal (38 g)

Calories	109	L-glutamine (mg) added	2000
Protein (g)	20	Creatine Monohydrate (mg)	1500
L-leucine (mg) added	1000	HMB (mg)	2000
L-isoleucine (mg) added	500	Total fat (g)	0.76
L-valine (mg) added	500	Total carbohydrate (g)	5.6

Supplements were provided to participants in the Sarcomeal group every two weeks. To ensure compliance, both the control and intervention groups had weekly visit schedules, with the same format. The control group completed all visits by telephone, while the intervention group alternated between telephone visits one week and in-person visits the following week, during which they returned empty sachets and received new medications. During each visit, whether by telephone or in person, the importance of adhering to the recommendations for exercise and following a protein-rich diet was emphasized. The only difference was that, during in-person visits, empty sachets were collected (to ensure adherence) and new medications were provided to the patients. Moreover, the occurrence of any side effects was asked from the intervention group in each visit. Blood samples and assessments of muscle parameters were conducted at both the initial and final visits (weeks 0 and 12).

### Outcomes

Three categories of outcomes were assessed:

The first category is muscle parameters and patient anthropometrics: skeletal muscle mass index (kg/m^2^) defined as the mass of appendicular skeletal muscles (arms and legs) relative to height (measured by DXA scan), handgrip strength (kg) defined as the maximum force exerted by the hand and forearm muscles when gripping (measured by digital dynamometer), gait speed (m/s) defined as the rate at which an individual walks (measured by a stopwatch), lean mass (g) defined as the total weight of the skeletal muscles in the body, excluding fat, bones, and other tissues (measured by DXA scan), percentage of fat defined as the proportion of an individual’s total body weight that is composed of fat tissue (measured by DXA scan), and BMI (kg/m2) defined as weight in kilograms divided by the square of height in meters (calculated using its formula). Moreover, weight (kg) was evaluated by having the individual stand on a calibrated scale, ensuring they were wearing minimal clothing and had removed any heavy items. Waist circumference (cm) was also measured at the midpoint between the lower margin of the last palpable rib and the top of the iliac crest using a non-stretchable tape. Calf circumference (cm) was measured at the widest part of the calf while the participant was standing with feet shoulder-width apart and weight evenly distributed, using a non-stretchable tape, ensuring the tape was snug but not compressing the skin. Finally, blood pressure was measured using a calibrated automatic sphygmomanometer, with participants seated and resting for at least 5 min prior to the measurement.

The second set of outcomes were blood parameters (all measured by blood sampling). Blood samples were collected after an overnight fast of at least 8 h. Venous blood was drawn from the antecubital vein using a sterile technique. The undertaken tests were:

- Sugar Profile: Glycated hemoglobin (HbA1c) and fasting blood sugar (FBS) levels were measured to assess long-term and short-term blood glucose control, respectively.
- Inflammatory Markers: High-sensitivity C-reactive protein (hs-CRP) was measured as a marker of systemic inflammation.
- Lipid Profile: Serum levels of triglycerides, total cholesterol, and high-density lipoprotein (HDL) cholesterol were assessed to evaluate lipid metabolism.
- Liver and Kidney Parameters: Serum glutamic-oxaloacetic transaminase (SGOT, also known as AST), serum glutamic-pyruvic transaminase (SGPT, also known as ALT), and creatinine levels were measured to assess liver and kidney function.

The final set of outcomes were scores of Quality of life in sarcopenia using the SarQol questionnaire: The SarQoL questionnaire [19, 20] consists of 22 questions across seven domains: physical and mental health (5 questions), locomotion (9 questions), body composition (2 questions), functionality (4 questions), activities of daily living (ADLs) (2 questions), leisure activities (3 questions), and fears (2 questions). The total score as well as separate scores for each domain were collected and analyzed.

Moreover, an assessment of the safety and tolerability of the study regimen was conducted among all participants who received at least one supplement. Any side effects associated with the Sarcomeal supplement were documented. Safety assessments encompassed a thorough review of participant medical histories, documentation of medication and nutritional supplement usage, and monitoring of adverse events via regular telephone calls throughout the intervention period and at each visit. While patients’ adherence to treatment was not defined as an outcome, it was recorded.

### Statistical analysis

Based on the study by Bo et al. [21], the average muscle strength changes for both intervention and control groups were extracted. With a power of 80% (β = 0.2) and a significance level of 0.05 (α = 0.05), the required sample size per group was calculated to be 25. To account for potential dropouts and non-cooperation, the sample size was increased by 20%, resulting in a final sample size of 30 participants per group.

For data analysis, descriptive and analytical statistics were conducted using SPSS 21 statistical software. The normality of variable distribution was assessed using the Kolmogorov-Smirnov and Shapiro-Wilk tests. For baseline variables, data are presented as frequencies for categorical data and mean and standard deviation (SD) for continuous variables and medians and interquartile ranges (IQRs) for non-normal data. A comparison of baseline variables was performed using a Chi-square test for categorical variables and independent t-tests for continuous variables. In instances of non-normality, equivalent non-parametric tests (Wilcoxon Signed-Rank Test and Mann-Whitney U Test) were applied. Participants were analyzed according to their respective groups, with all individuals included in the primary analysis (per protocol). A paired-sample t-test was used to compare within-group differences (pre- and post-treatment). The effect of intervention treatment (comparison of two groups) was evaluated by using an analysis of covariance (ANCOVA) with sex and age as covariates. A two-sided *P* < 0.05 was considered statistically significant.

## Results

### Patient enrollment and follow-up

**Fig. 1:**
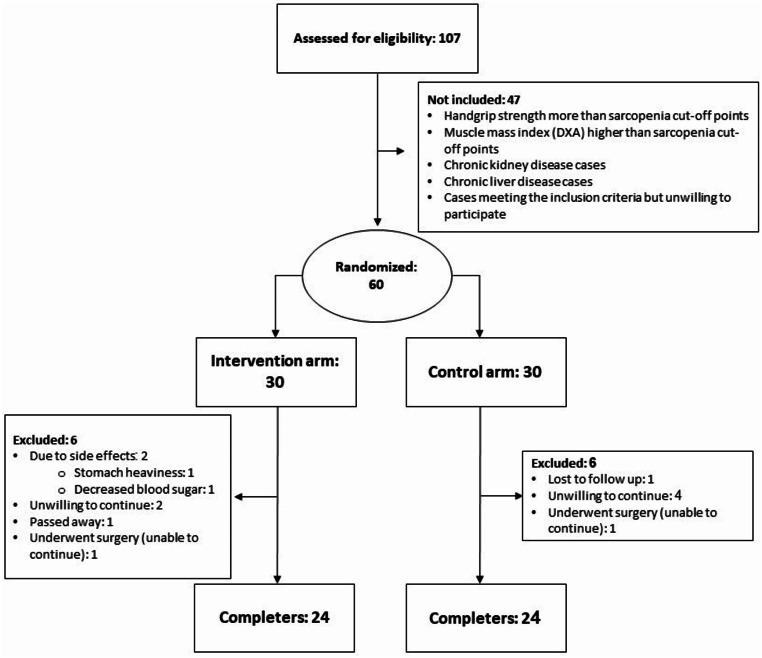
The flow diagram of participant screening, randomization, and follow-up

The flowchart of patient enrollment for this study is shown in Fig. 1. As can be seen, a total number of 107 patients were screened and evaluated for inclusion. While 47 patients were not included due to the defined criteria, 60 patients were randomized into the intervention and the control group. There were six patients excluded during the follow-up period in the control arm. Reasons were undergoing surgery (*n* = 1), unwillingness to continue (*n* = 4), and losing to follow-up (*n* = 1). In the meantime, there were also six patients excluded from the intervention group. Reasons included side effects (stomach heaviness [*n* = 1] and decreased blood sugar [*n* = 1]), unwillingness to continue (*n* = 2), passing away (*n* = 1), and undergoing surgery (*n* = 1). Finally, 24 participants in each group completed the 12-week study period.

### Patient demographics

The baseline demographic and clinical characteristics of the included patients (for all cases and each separate group) are reported in Table 2. The mean age of participants was 69.27 ± 5.69 years, with 62.5% of participants being men. The difference in mean age and percentage of men in the two groups was not significant. Baseline anthropometrics such as weight (60.70 ± 8.15 kg), BMI (22.23 ± 2.37 kg/m^2^), calf circumference (31.77 ± 2.03 cm), and waist circumference (93.87 ± 7.41 cm), as well as baseline systolic (120.94 ± 1.19 mmHg) and diastolic (89.06 ± 1.87 mmHg) blood pressure, did not significantly differ among the two groups.

**Table 2:** Baseline demographic and clinical characteristics of participants

Variable	Total(*n* = 48)	Intervention group(*n* = 24)	Control group (*n* = 24)	*P*-value
Age, years, mean (SD)	69.27 (5.69)	69.33 (5.69)	69.20 (5.82)	0.94
Sex, n men (%)	30 (62.5%)	16 (66.7%)	14 (58.3%)	0.65
Weight, kg, mean (SD)	60.70 (8.15)	61.29 (9.06)	60.13 (7.27)	0.62
BMI, kg/m^2^, mean (SD)	22.23 (2.37)	22.66 (2.38)	21.81 (2.31)	0.37
Calf circumference, cm, mean (SD)	31.77 (2.03)	32.0 (2.08)	31.54 (1.99)	0.44
Waist circumference, cm, mean (SD)	93.87 (7.41)	93.79 (8.16)	93.96 (6.75)	0.93
Systolic blood pressure, mmHg, mean (SD)	120.94 (1.19)	122.08 (1.53)	119.79 (1.84)	0.34
Diastolic blood pressure, mmHg, mean (SD)	89.06 (1.87)	91.25 (2.54)	86.87 (2.74)	0.25
Handgrip strength, kg, mean (SD)	19.08 (5.08)	19.12 (4.62)	19.06 (5.61)	0.97
SMI, kg/m^2^, mean (SD)	5.61 (0.72)	5.78 (0.64)	5.49(0.78)	0.59
Gait speed, m/s, mean (SD)	1.01 (0.27)	0.98 (0.26)	1.06 (0.29)	0.33
Percentage of Fat mass (%)	30.80(8.59)	30.09(7.47)	29.06(9.61)	0.58
Lean mass, kg, mean (SD)	38.56 (6.53)	39.52 (7.16)	38.89 (6.19)	0.90
Lean mass index, kg/m^2^, mean (SD)	14.02(1.29)	14.28(1.24)	13.87(1.30)	0.74
History of falling, n (%)	11 (22.9%)	7 (29.2%)	4 (16.7%)	0.21
History of osteoporosis, n (%)	6 (12.5%)	4 (16.7%)	2 (8.3%)	0.64
History of fractures, n (%)	15 (31.3%)	9 (37.5%)	6 (25.0%)	0.50
FBS, mg/dL, median (IQR)	138.0 (121.0-170.0)	132.0 (120.0-147.25)	144.5 (121.75-180.75)	0.17
HbA1C, %, mean (SD)	7.44 (1.17)	7.30 (1.10)	7.7 (1.30)	0.56
Triglyceride, mg/dL, median (IQR)	105.5 (83.5-132.75)	94.0 (82.25-127.75)	118.50 (88.25-178.25)	0.02
Total cholesterol, mg/dL median (IQR)	136.50 (109.50-163.50)	136.0 (109.75–163.50)	138.50 (105.75-167.75)	0.69
HDL, mg/dL, median (IQR)	45.30 (39.0–52.0)	46.30 (38.0–53.0)	45.0 (39.0–50.0)	0.88
CRP, mg/dL, median (IQR)	0.80 (0.48–2.02)	0.71 (0.41–2.52)	0.80 (0.50–2.02)	0.66
Creatinine, mg/dL, mean (SD)	0.93 (0.18)	0.99 (0.21)	0.87 (0.11)	0.03
AST, U/L, mean (SD)	21.40 (5.72)	20.4 (4.6)	22.4 (6.6)	0.20
ALT, U/L, mean (SD)	19.95 (7.54)	18.6 (5.6)	21.3 (9.0)	0.08

Data are presented as mean (SD); median (IQR) or number (percent). SD; Standard deviation; IQR: Interquartile Range BMI: Body mass index; SMI: Skeletal muscle mass index; FBS: Fasting blood sugar; HbA1C: hemoglobin A1C; HDL: high-density lipoprotein; CRP: C-reactive protein; AST: aspartate aminotransferase; ALT: alanine transaminase

Regarding muscle parameters, the mean handgrip strength was 19.08 ± 5.08 kg, the mean skeletal muscle mass index (SMI) was 5.61 ± 0.72 kg/m^2^, and the mean gait speed was 1.01 ± 0.27 m/s, not having any significant difference among the two groups. Fat mass, lean mass, and lean mass index did not also differ between the two groups at baseline. In the case of metabolic factors, except for mean triglycerides and mean creatinine, all other measurements did not significantly differ between the two groups at baseline (details in Table 2).

### Muscle parameters and patient anthropometrics

The changes in muscle parameters and anthropometrics of the two groups after 12 weeks are reported in Table 3. As can be seen, while the mean skeletal muscle index significantly improved over 12 weeks in the intervention group (change [95% CI]: 0.17 [0.016, 0.329] kg/m2; *p* < 0.05), the difference in the two groups did not reach statistical significance (mean difference [95% CI]: 0.15 [-0.05, 0.37] kg/m2; P: 0.14). The same results were also recorded for the handgrip strength; while the score significantly increased in the intervention group (change [95% CI]: 1.33 [0.256, 2.410] kg; *p* < 0.05), the difference in the two groups did not reach statistical significance (mean difference [95% CI]: 0.69 [-0.60, 1.98] kg/m2; P: 0.29). Gait speed, percentage of fat, and BMI did not significantly differ either within each group or between both groups after 12 weeks.

**Table 3:** Comparison of muscle parameters and anthropometrics in two groups after 12 weeks

Parameter	Intervention group ^a^					Control group				Intervention effect ^b^	
	Before	After	Within-group change ^c^	*P* -value		Before	After	Within group change ^c^	*P* value	Intervention effect (between-group difference) ^c^	*P* value
Skeletal muscle mass index (kg/m^2^)	5.78 ± 0.64	5.95 ± 0.66	**0.170** **(0.016**,** 0.329)**	**0.032**		5.49 ± 0.78	5.50 ± 0.76	0.015 (-0.141, 0.171)	0.84	0.15 (-0.052, 0.374)	0.14
Handgrip strength (kg)	19.12 ± 4.62	20.45 ± 4.74	**1.33** **(0.256**,** 2.410)**	**0.017**		19.06 ± 5.61	19.73 ± 5.59	0.67 (-0.046, 1.389)	0.06	0.69 (-0.605, 1.982)	0.29
Gait speed (m/s)	0.98 ± 0.26	1.04 ± 0.28	0.06 (-0.082, 0.209)	0.37		1.06 ± 0.29	0.97 ± 0.22	-0.08 (-0.183, 0.144)	0.09	0.14 (-0.034, 0.324)	0.10
Percentage of fat (%)	30.09 ± 7.47	29.77 ± 7.70	-0.32 (-1.203, 0.559)	0.45		29.06 ± 9.61	29.28 ± 9.51	0.25 (-0.455, 0.966)	0.45	-0.62 (-1.728, 0.489)	0.26
Lean mass (kg)	39.52 ± 7.16	40.47 ± 7.05	**0.94** **(0.367**,** 1.527)**	**0.003**		38.89 ± 6.19	38.14 ± 5.45	-0.75 (-1.573, 0.055)	0.06	**1.70 (0.749**,** 2.665)**	**0.001**
Lean mass index, kg/m^2^,mean	14.28 ± 1.24	14.64 ± 1.28	**0.35** **(-0.358**,** 0.877)**	**0.002**		13.87 ± 1.30	13.62 ± 1.18	-0.24 (-0.174, 0.013)	0.075	**0.62(0.287**,** 0.954)**	**0.001**
BMI (kg/m2)	22.66 ± 2.38	22.75 ± 2.34	0.08 (0.170, 0.343)	0.49		21.81 ± 2.31	21.67 ± 2.34	-0.13 (-0.319, 0.408)	0.12	0.23 (-0.046, 0.512)	0.10
Weight (kg)	61.29 ± 9.06	62.12 ± 8.59	0.82 (-0.142, 1.800)	0.09		60.13 ± 7.27	59.04 ± 7.09	**-1.08** **(-1.896**,** -0.269)**	**0.01**	**1.87 (0.654**,** 3.109)**	**0.003**
Waist circumference (cm)	93.79 ± 8.16	95.46 ± 8.09	**1.66** **(0.293**,** 3.040)**	**0.02**		93.96 ± 6.75	94.83 ± 5.65	0.87 (-1.297, 3.047)	0.41	0.78 (-1.693, 3.264)	0.52

^*a*^ *Significance level of the estimate of change at week 12 by using a paired T-test*

^*b*^ *Effect of intervention treatment (comparison of two groups) was evaluated by using an ANCOVA with sex and age as covariates*

^*c*^ *Values are means with upper and lower 95% CI bounds in parentheses*

Regarding lean mass, a significant increase was observed in the intervention group (change [95% CI]: 0.94 [0.367, 1.527] kg; *p* < 0.01). The difference in this increase also reached statistical significance compared to the control group (mean difference [95% CI]: 1.70 [0.749, 2.665] kg; *P* < 0.01). The same results were also observed for lean mass index (intervention effect: 0.62 [0.287, 0.954] kg/m^2^; *P* < 0.01). While participants in the intervention group did not experience any significant changes in their weight (change [95% CI]: 0.82 [-0.142, 1.8000] kg; p: 0.09), participants of the control group experienced a significant weight loss (change [95% CI]: -1.08 [-1.896, -0.269] kg; *p* < 0.05). The weight change difference reached statistical significance between the two groups (mean difference [95% CI]: 1.87 [0.654, 3.109] kg; *P* < 0.01).

**Fig. 2:**
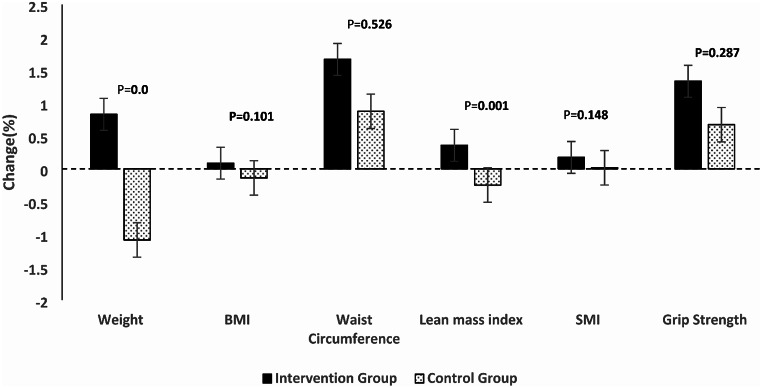
Percentage of change in each variable after a 12-week consumption of sarcomeal plus vitamin D

Finally, while waist circumference significantly increased in the intervention group (change [95% CI]: 1.66 [0.293, 3.040] cm; *p* < 0.05), the difference in this increase did not reach statistical significance when compared to the intervention group (mean difference [95% CI]: 0.78 [-1.693, 3.264] cm; p: 0.52). The percentage of change in each variable after a 12-week consumption of sarcomeal plus vitamin D supplement can be seen in Fig. 2.

### Blood parameters

The comparison of blood parameters both within and between the two groups after 12 weeks is reported in Table 4. As can be seen, the blood parameters of sugar profile, lipid profile, inflammatory markers, liver profile, and kidney profile were not significantly changed during the trial either within each of the groups or between the two groups. In the intervention group, the mean creatinine level had a change of 0.04 [-0.023, 0.110] mg/dL over the trial period (P: 0.18). This is while the control group had a change of 0.07 [-0.176, 0.316] mg/dL over the trial period (p: 0.55). The mean difference in the change of creatinine levels between the two groups was 2.69 [-9.961, 15.353] mg/dL (p: 0.66).

**Table 4:** The comparison of blood parameters in two groups after 12 weeks

Blood Parameter	Intervention group ^a^					Control group				Intervention effect ^b^	
	Before	After	Within-group change ^c^	*P* value		Before	After	Within-group change ^c^	*P* value	Intervention effect (between-group difference) ^c^	*P* value
FBS (mg/dL)	139.7 ± 33.1	154.3 ± 65.1	14.60 (-18.372, 47.572)	0.36		163.5 ± 52.19	174.5 ± 66.5	10.95 (-9.390, 31.290)	0.27	5.201 (-33.201, 43.603)	0.78
Triglyceride (mg/dL)	95.4 ± 32.6	103.2 ± 45.3	7.76 (-17.935, 33.465)	0.53		148.1 ± 98.8	134.4 ± 111.4	-13.75 (-42.493, 14.993)	0.32	20.01 (-18.341, 58.389)	0.29
Total cholesterol (mg/dL)	138.0 ± 37.7	135.1 ± 26.9	-2.90 (-21.381, 15.571)	0.74		142.9 ± 39.3	131.2 ± 31.4	-11.75 (-27.568, 4.068)	0.13	6.46 (-16.623, 29.545)	0.57
HDL-c (mg/dL)	48.6 ± 16.7	45.0 ± 13.3	-3.59 (-14.484, 7.303)	0.50		47.5 ± 11.2	46.4 ± 15.1	-1.10 (-8.142, 5.942)	0.74	-2.12 (-14.824, 10.574)	0.73
HbA1c (%)	7.3 ± 1.1	7.1 ± 1.2	-0.14 (-0.594, 0.307)	0.51		7.7 ± 1.3	7.8 ± 1.2	0.16 (-0.376, 0.696)	0.54	-0.28 (-0.988, 0.416)	0.41
CRP (mg/dL)	3.1 ± 6.9	4.7 ± 11.7	1.65 (-1.781, 5.081)	0.30		6.1 ± 19.7	1.2 ± 1.4	-4.92 (-15.945, 6.087)	0.35	5.29 (-7.389, 17.974)	0.39
Creatinine (mg/dL)	0.99 ± 0.21	1.0 ± 0.21	0.04 (-0.023, 0.110)	0.18		0.87 ± 0.11	0.94 ± 0.58	0.07 (-0.176, 0.316)	0.55	2.69 (-9.961, 15.353)	0.66
AST (U/L)	20.4 ± 4.6	22.0 ± 6.8	1.51 (-1.884, 4.906)	0.36		22.4 ± 6.6	20.6 ± 6.4	-1.75 (-5.578, 2.078)	0.35	2.99 (-2.183, 8.172)	0.24
ALT (U/L)	18.6 ± 5.6	18.1 ± 5.9	-0.50 (-3.773, 2.762)	0.74		21.3 ± 9.0	20.1 ± 8.1	-1.20 (-6.264, 3.864)	0.62	0.61 (-5.614, 6.854)	0.84

^*a*^ *Significance level of the estimate of change at week 12 by using a paired T-test*

^*b*^ *Effect of intervention treatment (comparison of two groups) was evaluated by using an ANCOVA with sex and age as covariates*

^*c*^ *Values are means with upper and lower 95% CI bounds in parentheses*

Regarding liver profile, the mean AST and ALT levels in the intervention group had a mean change of 1.51 [-1.884, 4.906] U/L (p: 0.36) and − 0.50 [-3.773, 2.762] U/L (p: 0.74), respectively. In the control group, the mean changes were − 1.75 [-5.578, 2.078] U/L (p: 0.35) and − 1.20 [-6.264, 3.864] U/L (p:0.62) for AST and ALT, respectively. The difference between these changes did not also reach statistical significance between the two groups. The mean differences in changes were 2.99 U/L (95% CI: [-2.18, 8.17]; p: 0.24) for AST and 0.61 U/L (95% CI: [-5.61, 6.85]; p: 0.84) for ALT. Details of other blood parameters and their changes can be found in Table 4.

### Quality of life in sarcopenia

The comparison of total SarQol scores along with the score of each domain is reported in Table 5. As can be seen, while the total score of SarQol was not significantly changed during the trial (change: 0.79 [-9.863, 11.455]; p: 0.87), participants of the control group had a significant decrease in the total score (change: -12.26 [-20.037, -4.501]; *p* < 0.01). This difference in the changes between the two groups reached statistical significance (mean difference [95% CI]: 13.04 [2.73, 23.34]; *P* < 0.05).

**Table 5:** The comparison of SarQol (quality of life in sarcopenia questionnaire) scores in different domains after 12 weeks

SarQol Domain	Intervention group ^a^		Control group		Intervention effect ^b^	
	Within-group change ^c^	*P* value	Within-group change ^c^	*P* value	Intervention effect (between-group difference)^c^	*P* value
Total score	0.79 (-9.863, 11.455)	0.87	**-12.26 (-20.037**,** -4.501)**	**0.004**	**13.04 (2.731**,** 23.342)**	**0.02**
Fears	0.00	-	0.00	-	-	-
Leisure	-5.20 (-15.042, 4.625)	0.28	-5.20 (-11.418, 1.001)	0.09	0.04 (-11.467, 11.542)	0.99
ADL	5.83 (-6.249, 17.916)	0.32	**-13.88 (-26.841**,** -0.935)**	**0.03**	**20.51 (5.794**,** 35.233)**	**0.004**
Functionality	4.98 (-7.577, 17.547)	0.42	-2.97 (-16.032, 10.079)	0.64	8.85 (-7.194, 24.904)	0.27
Body composition	13.76 (-1.271, 28.808)	0.07	-11.11 (-23.304, 1.082)	0.07	**25.08 (6.976**,** 43.205)**	**0.006**
Locomotion	-4.51 (-22.435, 13.408)	0.60	-9.37 (-28.891, 10.141)	0.33	5.16 (-17.792, 28.111)	0.65
Physical and mental health	3.32 (-10.984, 17.625)	0.62	**-11.40 (-21.029**,** -1.783)**	**0.02**	14.1 (-1.241, 29.456)	0.07

^*a*^ *Significance level of the estimate of change at week 12 by using a paired T-test*

^*b*^ *Effect of intervention treatment (comparison of two groups) was evaluated by using an ANCOVA with sex and age as covariates*

^*c*^ *Values are means with upper and lower 95% CI bounds in parentheses*

Scores of domains including leisure, functionality, fears, and locomotion did not significantly change either within or between groups. Regarding the activities of daily living (ADL) domain, while there was not a significant change in the mean score of the intervention group after 12 weeks (change: 5.83 [-6.249, 17.916]; p: 0.32), the control group faced a significant reduction in the score of this domain (change: -13.88 [-26.841, -0.935]; *p* < 0.05). This difference in score change among the two groups reached statistical significance (mean difference [95% CI]: 20.51 [5.79, 35.23]; *P* < 0.01). In the body composition domain, the mean scores were not significantly changed in both the intervention (change: 13.76 [-1.271, 28.808]; p: 0.07) and the control (change: -11.11 [-23.304, 1.082]; p: 0.07) group. However, there was a significant mean difference in the changes of the two groups (mean difference [95% CI]: 25.08 [6.97, 43.20]; *P* < 0.01).

Finally, although the mean score of the physical and mental health domain did not change in the intervention group (change: 3.32 [-10.984, 17.625]; p: 0.62), the control group had a significant decrease in this domain’s score (change: -11.40 [-21.029, -1.783]; *p* < 0.05). However, the mean difference of changes did not reach statistical significance (mean difference [95% CI]: 14.1 [-1.24, 29.45]; P: 0.07).

### Side effects

Table 6 shows the side effects reported for sarcomeal and their rate of occurrence among the patients. As can be seen, 50% of participants reported a loss of appetite following the consumption of sarcomeal sachets. Nearly 21% of patients experienced stomach heaviness and upset stomach. Four patients (16.7%) reported constipation and related it to the use of sarcomeal sachets. Interestingly, one patient experienced increased appetite (4.2%) and another one reported a decrease in blood sugar (4.2%) and related it to the consumption of sarcomeal. Finally, regarding adherence, 90% of the participants in the intervention group who completed the study adhered fully to the medication regimen.

**Table 6:** Side effects and their rate of occurrence

Side effect	Rate, *n* (%)
Loss of appetite	12 (50%)
Upset stomach	5 (20.8%)
Constipation	4 (16.7%)
Bloating	2 (8.3%)
Increased appetite	1 (4.2%)
Decreased blood sugar	1 (4.2%)

## Discussion

Our results demonstrate that a nutritional supplement containing whey protein, creatine, HMB, BCAAs, and glutamine, along with vitamin D yielded significant improvements in SMI, handgrip strength, and waist circumference in the intervention group. However, these differences did not reach statistical significance when compared to the control group. One possible explanation for this could be the duration of this trial; the trial duration of 12 weeks may not have been long enough to observe larger, more statistically significant changes. Muscle adaptations, particularly in sarcopenic individuals, may require more time to manifest in ways that create significant group differences. For example, in other trials, while other interventions such as 6 g/day of leucine [22] or “12.8 g of protein + 1.2 g leucine + 120 IU vitamin D” per day [23] were considered for nearly 12 weeks, they did not also reach significant intervention effect in the case of SMI and handgrip strength. However, while Bo et al. [21] used the daily dose of “22 g whey protein + 700IU vitamin D + 100 mg vitamin E” for 6 months, the intervention effect in the case of SMI and handgrip strength reached statistical significance. Moreover, another possible reason for these results may be the presence of diabetes along with sarcopenia. Chronic inflammation and altered glucose and protein metabolism may lead to mitochondrial dysfunction in myocytes, which can further lead to lower muscle synthesis even when supplements are being used.

On the other hand, lean mass and lean mass index showed significant improvements both within the intervention group and when compared to the control. These outcomes mean that consumption of sarcomeal supplement plus vitamin D has yielded muscle synthesis, leading to a significant improvement in the total skeletal muscle mass of the body. However, since SMI measures only the appendicular skeletal muscles (the sum of muscle masses of the four limbs), it can be another reason why data on SMI did not reach statistical significance in the analysis. The intervention of this study may have had more effect on other skeletal muscles rather than the appendicular skeletal muscles. It may also be concluded that in improving muscle mass in sarcopenic patients, some attention should be paid to other skeletal muscles as well, not just appendicular muscles. This assumption is further supported by other studies [24, 25], which report that variables such as total muscle mass and total lean mass can be increased in sarcopenic patients, while no significant changes are observed in the appendicular skeletal muscles.

Participants in the intervention group maintained their weight, while those in the control group experienced significant weight loss, resulting in a statistically significant difference between the two groups. The weight loss observed in the control group may be attributed to the progressive nature of the disease [26, 27], which can lead to a catabolic state and weight reduction, particularly in individuals with metabolic disorders like diabetes. In contrast, the maintenance of weight in the intervention group suggests that a nutritional supplement containing whey protein, creatine, HMB, BCAAs, and glutamine, along with vitamin D may have played a role in preserving lean mass, which aligns with the observed increase in lean mass and the stable fat percentage in both groups. Previous studies have demonstrated that interventions targeting lean mass preservation can mitigate weight loss [28]. These findings suggest that this supplement may have contributed to the maintenance of lean mass in the intervention group.

While parameters of diabetes were not significantly changed during this trial, the efficacy of both whey protein and vitamin D supplementation in controlling diabetes-related factors shows considerable inconsistency across other studies. In the case of whey protein, some studies report positive effects, such as improvements in postprandial glycemia, insulin secretion, and insulin resistance, particularly in well-controlled type 2 diabetes or overweight individuals at risk for diabetes [29, 30]. However, other studies, including meta-analyses [31, 32], highlight the lack of conclusive evidence on its long-term benefits, optimal dosages, and effects in lower-risk populations. Similarly, while some research supports the idea that vitamin D supplementation can reduce HbA1c levels in individuals with vitamin D deficiency or lower body mass index [33, 34], other trials fail to show significant improvements in glycemic control, particularly in the general diabetic population [35]. Both whey protein and vitamin D exhibit potential in improving certain diabetic factors, but inconsistent results and gaps in study designs underscore the need for further, more targeted research to better understand their effects on diabetes management.

Interestingly, blood parameters—including sugar, lipid, inflammatory markers, liver, and kidney profiles—remained unchanged in both groups, indicating that this mixture of nutritional supplements did not have any notable adverse effects on metabolic health. This is significant, as maintaining metabolic stability is essential in elderly and sarcopenic populations, where comorbidities like diabetes or cardiovascular disease are common. The lack of change in inflammatory markers, however, suggests that while Sarcomeal may improve muscle outcomes, it might not directly impact systemic inflammation, or that the trial duration was insufficient to detect such changes.

Regarding quality of life (SarQol) measures, the control group showed a significant decline, which may reflect the natural progression of sarcopenia and associated functional impairments. While this result may seem unexpected, there are other studies in the literature similarly showing the decline in the quality of life in the same period of time [36, 37]. The difference between groups was statistically significant, supporting the hypothesis that a blend of whey protein, creatine, HMB, BCAAs, and glutamine helps mitigate declines in overall quality of life. Specifically, the ADL domain showed no significant change in the intervention group, whereas the control group experienced a significant decrease. This suggests that this supplement may help preserve physical function, possibly through its effects on muscle mass and strength, which are closely linked to daily functional capacity in older adults. In the body composition domain, although there were no significant changes in either group, the significant difference observed between the groups could be indicative of subtle positive shifts in body composition in the intervention group, which might become more pronounced with a longer intervention period. Finally, while physical and mental health scores did not change in the intervention group, the control group experienced a significant decline, though the difference between groups was not statistically significant. This finding suggests that while Sarcomeal might have a protective effect on mental and physical health, a longer trial or larger sample size may be necessary to fully elucidate these benefits.

One important aspect that is essential to acknowledge is that while the administration of Sarcomeal sachets, which include BCAAs, has demonstrated benefits in improving muscle parameters in diabetic sarcopenia patients, there are some risks associated with BCAA supplementation in this population. Studies have indicated that serum BCAA concentrations are often elevated in diabetic patients [38], which can lead to adverse effects such as impaired lipid and glucose metabolism and insulin resistance [39]. That is why studies suggest a BCAA-restricted diet in diabetic patients [40]. However, managing diabetic sarcopenia presents additional complexities, as BCAAs also offer significant benefits. That is why future research should continue to explore the balance between the benefits of BCAA supplementation for muscle health and the metabolic risks in diabetic patients, potentially through longer-term studies and broader patient populations.

Sarcopenia among individuals with diabetes presents a multifaceted challenge and is influenced by various factors such as insulin resistance, inflammation, oxidative stress, and hormonal changes [41]. Addressing this condition often involves considering nutritional interventions aimed at preventing muscle loss and preserving physical function in diabetic individuals [42]. Although the specific medications tailored for DS remain unclear, the pivotal role of nutrition in improving this condition is evident. Notable nutrients proven to be beneficial in DS encompass dietary or whey protein, omega-3 fatty acids, vitamin D, creatine, and certain antioxidants [43, 44].

The efficacy of whey protein in addressing DS is attributed to its rich content of essential amino acids and leucine, which promote muscle synthesis. Notably, a randomized controlled trial [45] conducted over 13 weeks demonstrated that daily consumption of whey protein supplementation led to improvements in muscle mass and strength among older adults with type 2 diabetes. Several other randomized controlled trials have also suggested the potential benefits of whey protein supplementation in halting the progression of sarcopenia in this population [46]. Moreover, a recent systematic review and meta-analysis by Chiang et al. (2022) [31] accumulated these findings, indicating that whey protein supplementation could also enhance glycemic control in individuals with type 2 diabetes. Nonetheless, the optimal dosage and duration of whey protein intake for preventing muscle loss in DS remain uncertain. Moreover, it’s essential to consider potential side effects, such as gastrointestinal discomfort and kidney problems, before recommending whey protein supplementation as a nutritional intervention for DS.

While creatine supplementation has demonstrated efficacy in enhancing muscle mass and strength among healthy individuals and athletes [47], its effects on individuals with DS are yet to be fully understood. A review by Candow et al. (2021) [48] showed that creatine supplementation could serve as a potential nutritional strategy for improving sarcopenia, suggesting that it may increase muscle mass and strength through mechanisms such as heightened protein synthesis, diminished protein breakdown, and augmented satellite cell activation. Additionally, creatine’s purported antioxidant properties may confer protection against muscle damage induced by oxidative stress as well [49]. Nevertheless, the evidence supporting the use of creatine supplementation for improving muscle mass and function specifically in individuals with type 2 diabetes and sarcopenia remains limited. Further investigation is warranted to ascertain the optimal dosage and duration of creatine supplementation for this population.

The precise mechanisms underlying the effects of BCAA and HMB supplements on skeletal muscle mass and function in individuals with diabetes are not yet fully elucidated. However, a study indicated that BCAA supplementation may enhance muscle mass and function among older adults with sarcopenia [50]. Additionally, a systematic review [51] highlighted that HMB supplementation may effectively prevent metabolic and physical complications associated with aging, including frailty, dynapenia, sarcopenia, and sarcopenic obesity, while also preserving health, functional capacity, and strength in older individuals. Furthermore, vitamin D has been associated with improved muscle strength and function, with some evidence suggesting that its supplementation may amplify the benefits of whey protein on muscle mass and function [52, 53]. Consequently, vitamin D supplementation is also considered effective in addressing muscle dystrophies, including diabetic sarcopenia.

This clinical trial offers several strengths: Firstly, this study used a comprehensive approach for choosing the intervention, including nearly all of the components that are being used in muscle synthesis. Even though vitamin D is not included in the sarcomeal sachet, it was included in the intervention and was provided for patients. Moreover, the detailed assessment of various outcomes including muscle parameters, anthropometrics, blood parameters, and quality of life measures offers a broad evaluation of the supplement’s impact, providing insights into both physical and metabolic conditions. Finally, while former studies have concentrated on sarcopenia itself, this study emphasized diabetic sarcopenia, which is a prevalent comorbidity in diabetic patients.

Despite its strengths, the study has several limitations. The open-label design, while practical, may introduce unconscious biases, as both participants and researchers were aware of the treatment allocation. The lack of significant changes in some outcomes, such as blood parameters and certain quality of life domains, suggests that the impact of Sarcomeal may require a more extended period to be fully realized. Another limitation of this study is the variability in adherence to the recommended exercise and protein intake guidelines among participants. Although efforts were made to encourage compliance through regular follow-up, individual adherence could still vary. The Final limitation of this study is the use of a per-protocol analysis instead of Intention-to-treat (ITT). ITT was not feasible because key outcomes required final assessments that were missed by non-adherent patients.

## Conclusion

In conclusion, our randomized controlled clinical trial demonstrated promising effects of the synergistic blend of whey protein, creatine, BCAAs, glutamine, and HMB on lean mass, lean mass index, and overall quality of life in sarcopenic patients. The significant increase in lean muscle mass, coupled with the preservation of physical function and activities of daily living, suggests that this supplement may play a key role in countering the muscle loss and functional decline typical of sarcopenia. The maintenance of weight and stabilization of metabolic health in the intervention group further underscores the potential of this supplement as an effective nutritional strategy to support muscle health and overall well-being in this population. These results highlight the value of targeted supplementation in promoting muscle preservation, physical function, and quality of life in older adults facing sarcopenia.

## Data Availability

No datasets were generated or analysed during the current study.
